# Breast Cancer Survival in Germany: A Population-Based High Resolution Study from Saarland

**DOI:** 10.1371/journal.pone.0070680

**Published:** 2013-07-31

**Authors:** Bernd Holleczek, Lina Jansen, Hermann Brenner

**Affiliations:** 1 Saarland Cancer Registry, Saarbrücken, Germany; 2 Division of Clinical Epidemiology and Aging Research, German Cancer Research Center, Heidelberg, Germany; Queensland University of Technology, Australia

## Abstract

Population-based survival studies of breast cancer patients are commonly restricted to age- and stage-specific analyses. This study from Germany aimed at extending available population-based survival data on further prognostic cancer characteristics such as tumor grade, hormone receptor status and human epidermal growth factor receptor type 2 (HER2/neu) expression. Data from the population-based Saarland Cancer Registry including female patients diagnosed with invasive breast cancer between 2000 and 2009 were included. Period analysis methodology and regression modelling were used to obtain estimates of 5-year relative survival and tumor related excess risks in 2005-2009. Overall age standardized 5-year relative survival was 83%. In addition to age and stage, tumor grade and hormone receptor status were independent predictors of 5-year relative survival. Detailed analyses by age, stage, morphology, tumor grade, hormone receptor status and HER2/neu expression consistently revealed lower survival of patients with high grade, hormone receptor negative or HER2/neu positive cancers and patients aged 70 years or older. This high resolution study extends available population-based survival data of breast cancer patients. Particular efforts should be made to overcome the persisting large survival deficits, which were observed for elderly patients in all clinical subgroups.

## Introduction

Breast cancer (BRC) is the most frequent cancer among women with a lifetime risk of up to 12% and a lifetime risk of death of up to 5%[1,2]. It was estimated to cause approximately 421,000 new cases and 129,000 deaths in Europe alone in 2008[3]. Within the past two decades, BRC mortality has gradually decreased as a result of increased early detection, mass screening, and therapeutic improvements[4,5,6].

Age and stage of disease at diagnosis are the most important prognostic factors. In observational studies, their distribution is crucial to understand and account for effects of early detection, mass screening and cancer treatment and to understand differences in cancer survival observed between health care systems or trends over time. Morphology, tumor grade, presence of hormone receptors (HR) and expression of the human epidermal growth factor receptor type 2 (HER2/neu) are further important prognostic factors of BRC with regard to disease recurrence and survival[7,8,9,10,11,12,13]. However, population-based data on these factors are scant, as survival studies from population-based cancer registries commonly are restricted to age and stage[14,15,16,17,18].

This high resolution study from Germany aims at extending available population-based survival data, which were mostly restricted to age- and stage-specific estimates in the past by further prognostic cancer characteristics such as tumor grade, HR status and HER2/neu expression.

## Material and Methods

This study used data from the Saarland Cancer Registry, which covers the federal state of Saarland in South-Western Germany with a population of approximately 1.04 million inhabitants in 2006. The registry collects information on invasive and in situ neoplasms since 1968 through notifications from pathology laboratories, hospitals, radiotherapy departments, outpatient clinics, screening programmes, and general practitioners. In Saarland, the notification of invasive and in situ tumors is mandatory by law for any physician, and the proportion of incident cancer cases included in the registry database is regularly estimated to be higher than 95%[19,20,21].

The study database included 8571 female patients with invasive BRC (ICD-10 code: C50) diagnosed between 2000 and 2009 and aged 15 years or older. Patients with a previous invasive BRC were excluded.

Mortality follow-up based on death certificates from state health authorities was available until end of 2009. Additionally, linkage of patients assumed alive by end of 2009 with the central population registry was performed to capture out-migration and missed deaths of the included cancer patients. Patients having left the covered area contributed survival time until removal. Patients for whom linkage with the population registry was not possible (e.g. due to erroneous personal identifiers) and without follow-up information were categorized as "no follow-up available" and excluded from the survival analyses. Patients with death certificate only (DCO) notified tumors were further excluded from the survival analyses.

In addition to routine tumor information collected by the registry (including month and year of diagnosis, sex, age at diagnosis, cancer site and morphology according to ICD-O3 [22], tumor grade, TNM information and stage [23,24], most valid basis of diagnosis, month and year of end of follow-up, vital status and cause of death), data on HR status and HER2/neu expression were obtained by means of additional reports from pathology departments and case summaries from providers of cancer care. Furthermore, data were collected at source by registry staff. For this purpose, eligible patients were identified in the registry database. Based on a standardized extraction protocol, data were extracted from available routine medical documentation by trained registrars, and standard procedures of quality control (with respect to accuracy, completeness and consistency of the extended data) were applied.

For the analyses, three age categories were used: 15-49, 50-69 and 70 years or older. Stage was classified as “localized” (T1-3N0M0), “regional or local spread” (T1-3N+M0 or T4M0), “distant” (M1) and “unknown”, according to the European Network of Cancer Registries recommendations[25]. For patients without surgery treatment and pathologic stage, clinical extent of disease was used. Grading included the categories “low” (G1), “intermediate” (G2), “high” (G3/4), and “missing” according to the WHO scheme. HR status (based on immunohistochemistry; for two patients, biochemistry was used for quantitative measurements) was classified as “positive” (both estrogen and progesterone receptor positive), “mixed” (either estrogen or progesterone receptor positive), “negative” (both estrogen and progesterone receptor negative), and “missing”. The categories of HER2/neu expression were “positive” (including borderline), “negative” and “missing”. Univariate description of the patients and tumor characteristics was derived for the calendar intervals 2000-2004 and 2005-2009, respectively.

Relative survival (RS) which quantifies excess mortality due to the cancer (capturing both direct and indirect mortality) is derived as ratio of survival observed for the cancer patients and survival expected for a sex-age- and calendar time-matched group of individuals with average risk of death from all causes of the underlying population (then, a RS of 100% results if the observed mortality of the patients is equal to the expected mortality according to the used life tables)[26]. The Ederer II method was used for deriving expected survival estimates[27]. Details on the generation of the used life tables may be found elsewhere[28].

Period analysis was used to obtain up-to-date estimates of 5-year RS. The method uses survival experience observed in a specified calendar period (typically the most recent calendar period, for which incidence and mortality follow-up are available) and, in addition to right censoring, survival observations are left truncated at the beginning of the calendar period[29]. Extensive empirical evaluation has shown that period estimates closely predict survival later observed for the patients diagnosed in the respective calendar period[29,30,31,32].

Period estimates of 5-year RS were derived for the calendar period 2005-2009 by age and tumor characteristics. Reported standard errors are based on the Greenwood method[33]. Age standardized survival was derived as weighted average of age group-specific survival using the International Cancer Survival Standards (ICSS)[34].

To derive estimates of relative excess risk of death (RER) and for statistical significance testing, model-based period analysis was used as previously described[35,36]. Based on an additive hazards model, RER quantifies the relative cancer related excess mortality between the specific “exposed” groups of patients (defined by age, stage or other characteristics) compared to an “unexposed” reference group of matched persons from the general population (the RER can also be interpreted as excess hazard ratio)[37,38].

To investigate effects of age and tumor characteristics on 5-year RS and RER, explanatory variables of categorical type were included into the linear predictor of the logarithm of the excess number of deaths[38].

Two types of models were derived: regression models adjusting for age and stage effects (I) and "complete" models including tumor morphology, tumor grade, HR status and HER2/neu expression in addition to age and stage (II). For the RER estimates, 95% confidence intervals (CI) were derived. The reported p-values are based on significance tests on the inclusion of the respective variable into the models.

To overcome loss of information and potential bias from missing information on tumor characteristics in the estimates of RS and RER, multiple imputation was used to derive completed datasets. Based on a missing at random (MAR) assumption, multiple imputation by chained equations, a method closely related to Gibbs sampling, was used according to a recently proposed approach for models of RS[39]. Based on information on age and stage at diagnosis, tumor grade, HR status, HER2/neu expression, follow-up duration (<6, 6-11, 12-35, 36-59, and >=60 months, respectively), vital status and cause of death (alive, death from BRC, death from other causes), multinomial logit models were derived for imputing missing data of a variable given the values of the other variables. The chosen imputation model assumes incompleteness of information to be independent within each stratum of the included model variables (such as age, tumor characteristics, follow-up duration, vital status) and adjusts for differences in the completeness of information between subgroups of patients with regard to tumor characteristics and duration of the disease. After 10 initial iterations (convergence of the models was assessed visually), 10 completed datasets were sampled. For each completed dataset, conventional and modelled period analysis was performed and combined estimates of 5-year RS, RER and corresponding standard errors were derived according to Rubin’s method[40]. Overall p-values of tests for differences between nested models were derived from likelihood ratio Chi-squared statistics (based on log-likelihood functions of the respective models)[41,42].

In addition to estimates of RS and RER presented in the main text, an additional table with observed (all cause) survival and excess risks of death is presented in Appendix S1 along with a short methodological note.

The R Language and Environment for Statistical Computing (release 2.11.1) [43] and the ”periodR” package (release 1.0-6) were used for data preparation, multiple imputation, survival estimation, and modelling[44,45].

## Results

Table 1 presents patient and tumor characteristics for the calendar periods 2000-2004 and 2005-2009, respectively. Overall, 8571 patients were included in this study. Mean age at diagnosis was 63.4 years. Information on tumor stage, morphology, tumor grade, HR status and HER2/neu expression were available for 84%, 97%, 94%, 86% and 71% of the patients, respectively. The proportion of patients with information on HER2/neu status increased from 60% in 2000-2004 to 81% in the second half of the study period. In 2005-2009, 50% of the patients had localized tumors, 42% had locally or regionally advanced BRC and 8% presented with distant metastases. Most frequently, the tumors were invasive ductal carcinomas (71%), of intermediate grade (68%), HR positive (72%), and HER2/neu negative (76%). The overall proportions of DCO notified cancers and patients without follow-up information were less than 2% each.

**Table 1:** Characteristics of patients with invasive breast cancer (ICD-10: C50) diagnosed in Saarland in 2000-2009.

Characteristic	Category	2000-2004		2005-2009	
		n	%	n	%
Overall		4147		4424	
Age	15-49	754	18.2	785	17.7
	50-69	1973	47.6	2098	47.4
	> = 70	1420	34.2	1541	34.8
Stage	available	3574	86.2	3642	82.3
	localized^a^	1817	50.8	1823	50.1
	regionally/locally advanced^a^	1424	39.8	1519	41.7
	distant^a^	333	9.3	300	8.2
Microscopic confirmation		4031	97.2	4312	97.5
Morphology	available	4031	97.2	4305	97.3
	invasive ductal^a^	2710	67.2	3041	70.7
	invasive lobular^a^	563	14.0	656	15.2
	mixed type^a^	352	8.7	279	6.5
	other^a^,^b^	406	10.1	329	7.6
Grading	available	3856	93.0	4201	95.0
	low^a^	277	7.2	304	7.2
	intermediate^a^	2273	58.9	2872	68.4
	high^a^	1306	33.9	1025	24.4
Hormone receptor status	available	3620	87.3	3720	84.1
	positive (ER+ PgR+)^a^	2474	68.3	2688	72.3
	mixed (ER+ or PgR+)^a^	531	14.7	451	12.1
	negative (ER-PgR-)^a^	615	17.0	581	15.6
HER2/neu	available	2505	60.4	3598	81.3
	positive^a^,^c^	625	25.0	866	24.1
	negative^a^	1880	75.0	2732	75.9
Death certificate only notified		67	1.6	100	2.3
No follow-up available		79	1.9	48	1.1

NB: ER = estrogen receptor; PgR = progesterone receptor; ^a^ proportions among patients with available information; ^b^ including mucinous (195) and tubular (109) carcinoma, M. Paget (57), inflammatory carcinoma (14), other specified (253) and non-specified (107) carcinoma; ^c^ including 401 tumors with borderline expression

The proportion of patients with clinical information on tumor stage only increased with age and extent of disease. Whereas the proportion of these patients was below 1% among those aged 15-49 years and those with localized BRC, it increased to 6.5% and 7.0% among patients aged 70 years or those with metastasized BRC, respectively, in 2005-2009 (data not shown).

Table 2 shows 5-year RS of BRC patients and RER by age and tumor characteristics. Overall age standardized 5-year RS of BRC patients was 83%. Survival decreased from 89% for patients aged 15-49 years and 88% for patients aged 50-69 years (RER: 1.20, 95%-CI: 0.91-1.58) to 77% for those aged 70+ years (2.02, 1.50-2.71). Age standardized 5-year RS was 98% for patients with localized BRC, 80% for those with locally or regionally spread BRC (5.63, 3.65-8.69) and 22% (44.15, 28.53-68.32) for patients presenting with distant metastases.

**Table 2:** Five-year relative survival and relative excess risk of death of female patients from Saarland with invasive breast cancer (ICD-10 code: C50) estimated for the calendar period 2005-2009 by age and tumor characteristics based on completed datasets.

Characteristic	Category	RS^a^	SE	RER^b^	95% CI	p-value	RER^c^,^d^	95% CI	p-value
Overall		83.2	0.9						
Age	15-49	89.1	1.2	1.00	REF		1.00	REF	
	50-69	88.0	0.9	1.14	0.86-1.49		1.20	0.91-1.58	
	70+	76.7	1.8	1.86	1.39-2.49	<0.001	2.02	1.50-2.71	<0.001
Stage	localized	97.8	1.3	1.00	REF		1.00	REF	
	regionally/locally advanced	79.7	1.5	7.01	4.22-11.66		5.63	3.65-8.69	
	distant	22.1	2.6	55.96	33.49-93.52	<0.001	44.15	28.53-68.32	<0.001
Morphology^d^	invasive ductal	82.5	1.1	1.00	REF		1.00	REF	
	invasive lobular	82.2	2.4	0.90	0.69-1.18		1.03	0.78-1.36	
	mixed type	93.2	3.0	0.65	0.41-1.01		0.83	0.54-1.30	
	other	80.5	2.8	1.22	0.86-1.72	0.083	1.07	0.76-1.51	0.790
Tumor grade^d^	low	101.2	3.2	1.00	REF		1.00	REF	
	intermediate	86.1	1.1	4.54	0.92-22.43		4.95	0.80-30.52	
	high	72.5	1.8	8.92	1.81-44.06	<0.001	7.11	1.15-44.03	<0.001
Hormone receptor status^d^	positive (ER+ PgR+)	87.7	1.0	1.00	REF		1.00	REF	
	mixed (ER+ or PgR+)	81.8	2.6	1.76	1.30-2.39		1.70	1.25-2.32	
	negative (ER-PgR-)	65.3	2.4	3.48	2.81-4.31	<0.001	2.92	2.31-3.70	<0.001
HER2/neu expression^d^	negative	85.0	1.0	1.00	REF		1.00	REF	
	positive	78.5	2.0	1.32	1.05-1.65	0.022	0.93	0.75-1.17	0.546

NB: RS: point estimate of 5-year relative survival; SE: standard error of RS; RER: relative excess risk (of death); CI: confidence interval; ER: estrogen receptor; PgR: progesterone receptor; REF: reference group; ^a^ except for age group-specific estimates, age standardized estimates were derived using the ICSS weights; ^b^ adjusted for age and stage; ^c^ adjusted for age, stage, morphology, tumor grade, hormone receptor status and HER2/neu expression ("complete" model); ^d^ cases without microscopic verification were excluded; the survival estimates were derived from 10 completed datasets using multiple imputation

Age standardized 5-year RS of patients with invasive ductal carcinomas was 83%. For patients with lobular carcinomas and carcinomas of mixed type, 5-year RS was 82% (RER: 1.03; 95%-CI: 0.78-1.36) and 93% (0.83, 0.54-1.30), respectively. Five-year RS was 101%, 86% (4.95, 0.80-30.52) and 73% (7.11, 1.15-44.03) for patients with low, intermediate and high grade tumors, respectively. Patients with HR positive, mixed, and negative tumors had age-standardized 5-year RS of 88%, 82% (RER: 1.70, 95%-CI: 1.25-2.32) and 65% (2.92, 2.31-3.70), respectively. For patients with HER2/neu negative tumors, 5-year RS was 85%, compared to 79% for patients with HER2/neu positive tumors (0.93, 0.75-1.17).

In addition to age and stage, tumor grade and HR status were independent predictors of 5-year RS and significantly improved the fit of the regression models (p-values: each <0.001). Overall, regression models adjusting for age and stage only provided quite similar results compared to the "complete" model. Solely the (rather small) effect of HER2/neu expression on RER decreased from 1.32 (p-value: 0.022) to 0.93 (p-value: 0.546), if further predictors were included into the model.

Table 3 presents 5-year RS by tumor morphology, age and stage. As in the combined analysis, RS of patients with carcinomas of mixed type was higher compared to invasive ductal or lobular carcinoma among the different categories of age and stage. This difference was particularly pronounced among elderly patients (98% vs. 75% and 77%) and patients with locally or regionally advanced BRC (88% vs. 79% and 78%, respectively). Survival of elderly patients with invasive ductal or invasive lobular carcinomas was inferior compared to patients aged 15-69 years. Survival of patients with metastasized BRC was quite similar for patients with tumors of invasive ductal, lobular and mixed type.

**Table 3:** Five-year relative survival of female patients from Saarland with invasive breast cancer (ICD-10 code: C50) by tumor morphology and stratified by age and stage estimated for the calendar period 2005-2009 based on completed datasets.

Morphology	overall			age				stage					
				15-69 years		70+ years		localized		locally/regionally advanced		distant metastasis	
	RS^a^	SE		RS	SE	RS	SE	RS^a^	SE	RS^a^	SE	RS^a^	SE
invasive ductal	82.5	1.1		88.4	0.8	74.9	2.3	96.8	1.7	79.2	1.8	21.8	2.2
invasive lobular	82.2	2.4		87.2	1.9	77.1	4.7	98.8	3.3	77.6	3.7	24.6	6.0
mixed type	93.2	3.0		90.6	2.3	97.7	5.9	104.5	3.7	87.7	5.3	25.1	13.0
other	83.0	2.9		87.4	2.7	76.0	5.7	97.4	3.4	80.4	5.9	7.4	6.5

NB: RS = point estimate of 5-year relative survival; SE = standard error of RS; ^a^ age standardized estimates were derived using the ICSS weights; cases without microscopic verification were excluded; the survival estimates were derived from 10 completed datasets using multiple imputation

Table 4 shows 5-year RS by tumor grade, age and stage. The gradient between low and high grade tumors observed in the combined analysis was particularly pronounced among patients aged 70 years or older (low grade: 99%, intermediate grade: 79%, high grade: 65%), patients with locally/regionally spread tumors (98% vs. 85% and 69%) and metastasized BRC (low grade: 24% vs. high grade: 8%).

**Table 4:** Five-year relative survival of female patients from Saarland with invasive breast cancer (ICD-10 code: C50) by tumor grade and stratified by age and stage estimated for the calendar period 2005-2009 based on completed datasets.

Grading	overall			age				stage					
				15-69 years		70+ years		localized		locally/regionally advanced		distant metastasis	
	RS^a^	SE		RS	SE	RS	SE	RS^a^	SE	RS^a^	SE	RS^a^	SE
low	101.2	3.2		101.4	1.2	99.2	6.2	103.7	3.6	98.2	8.8	24.0	13.3
intermediate	86.1	1.1		91.2	0.8	79.2	2.3	98.4	1.6	84.5	1.9	25.5	3.8
high	72.5	1.8		79.4	1.5	64.5	3.7	92.8	3.1	69.0	2.6	7.9	3.7

NB: RS = point estimate of 5-year relative survival; SE = standard error of RS; ^a^ age standardized estimates were derived using the ICSS weights; cases without microscopic verification were excluded; the survival estimates were derived from 10 completed datasets using multiple imputation

Five-year RS of BRC patients by HR status, age and tumor stage are presented in Table 5. The stratified analyses closely matched the pattern observed in the combined analysis. Again, pronounced survival differences between HR positive, mixed and negative tumors were seen for patients aged 70+ years (HR positive: 81%, mixed: 75%, negative: 56%), and those with locally/regionally advanced tumors (HR positive: 86%, mixed: 74%, negative: 58%) and metastasized BRC (HR positive: 30%, mixed: 16%, negative: 8%, respectively).

**Table 5:** Five-year relative survival of female patients from Saarland with invasive breast cancer (ICD-10 code: C50) by hormone receptor status and stratified by age and stage estimated for the calendar period 2005-2009 based on completed datasets.

Hormone receptor status	overall			age				stage					
				15-69 years		70+ years		localized		locally/regionally advanced		distant metastasis	
	RS^a^	SE		RS	SE	RS	SE	RS^a^	SE	RS^a^	SE	RS^a^	SE
positive (ER+ PgR+)	87.7	1.0		92.8	0.7	81.2	2.2	99.3	1.5	86.0	1.7	29.8	3.5
mixed (ER+ or PgR+)	81.8	2.6		86.0	2.2	75.4	5.2	101.3	3.5	74.1	4.4	16.3	7.3
negative (ER-PgR-)	65.3	2.4		73.4	2.1	55.8	5.0	86.7	4.4	57.7	4.3	8.2	3.6

NB: RS = point estimate of 5-year relative survival; SE = standard error of RS; ER: estrogen receptor; PgR: progesterone receptor; ^a^ age standardized estimates were derived using the ICSS weights; cases without microscopic verification were excluded; the survival estimates were derived from 10 completed datasets using multiple imputation

Table 6 presents estimated 5-year RS by HER2/neu expression, age and tumor stage. Overall and age specific survival of patients with tumors with HER2/neu expression was lower compared to patients without HER2/neu expressed tumors. The difference in 5-year RS between HER2/neu negative and positive tumors was approximately 6% units in both age categories. Age standardized 5-year RS was quite similar in patients with localized BRC, but differences were observed for patients with locally or regionally spread BRC (81% vs. 76%) and among patients with metastasized disease (25% vs. 18%).

**Table 6:** Five-year relative survival of female patients from Saarland with invasive breast cancer (ICD-10 code: C50) by HER2/neu expression and stratified by age and stage estimated for the calendar period 2005-2009 based on completed datasets.

HER2/neu expression	overall			age				stage					
				15-69 years		70+ years		localized		locally/regionally advanced		distant metastasis	
	RS^a^	SE		RS	SE	RS	SE	RS^a^	SE	RS^a^	SE	RS^a^	SE
negative	85.0	1.0		89.9	0.8	78.5	2.2	98.1	1.4	81.2	1.7	24.6	3.4
positive	78.5	2.0		84.0	1.7	72.1	4.5	96.5	3.0	76.0	3.4	17.6	4.7

NB: RS = point estimate of 5-year relative survival; SE = standard error of RS; ^a^ age standardized estimates were derived using the ICSS weights; cases without microscopic verification were excluded; the survival estimates were derived from 10 completed datasets using multiple imputation

Estimates of 5-year observed (all cause) survival and excess risks of death with regard to age and tumor characteristics are shown in Table S1 in Appendix S1. In general, the patterns seen for estimates of observed survival and excess risks of death were similar to those seen for RS estimates. However, as observed survival includes mortality from any cause, these estimates are lower than estimates of RS (e.g. for subgroups of patients with little tumor related excess mortality, such as patients with localized disease or low grade tumors, observed 5-year survival was 85% and 87%, compared to RS of 98% and 101%).

## Discussion

This population-based study presents most recent survival of BRC patients according to age, stage and other major prognostic tumor characteristics including tumor grade, morphology, HR status and HER2/neu expression. Regression analysis revealed tumor grade and HR status as independent prognostic factors of cancer related excess mortality (e.g. 7.1-fold increased risk of cancer death of patients with high grade tumors compared to those with low grade tumors, 2.9-fold risk of BRC patients with HR negative tumors compared to those with HR positive tumors). Further analyses stratified by age and stage demonstrated substantial variations of survival by tumor grade, HR status and HER2/neu expression (e.g. 5-year RS of patients with metastsized BRC of HER2/neu expressed tumors was 18% compared to 25% if the tumors showed no HER2/neu expression).

This study extends available population-based data on BRC survival. To our knowledge, population-based data on 5-year RS according to tumor grade, HR status and HER2/neu expression are scant, as available studies were generally restricted to overall survival or included age and stage stratified analyses only[14,16,17,18]. For Germany, such detailed population-based BRC survival data have not been presented yet.

Compared to other European countries, Saarland is ranking middle in terms of overall BRC survival (5-year RS of women diagnosed 1995-99; Saarland: 82%, Europe: ranging from 79% in Slovenia to 88% in Iceland) [16] and observed trends since 2000 (increase of 5-year RS of women in 2000-04; Saarland +5% units to 83%, Estonia: +14% units to 72%, Geneva region: +2% units to 88%)[46]. For a sample of 4500 patients from 17 European regions, 5-year RS of BRC patients diagnosed in 1990-1992 was presented by tumor morphology and HR status[47]. The reported estimates were similar when compared with corresponding estimates derived from the database of this study (5-year RS of women diagnosed 1990-92 in different regions in Europe; invasive ductal carcinoma: 81%, HR positive: 90%, HR negative: 73%), although the patients included in this study were diagnosed more than a decade later.

A recent comparison of age- and stage-stratified survival trends between Germany and the United States showed similar survival among patients younger than 70 years (5-year RS in 2005-08: Saarland: 88%; US: 89%), but marked differences for elderly patients (Saarland: 75%; US: 86%)[17]. Inferior survival of elderly patients is commonly explained by co-morbidity and differences in the delivery of cancer care[48,49,50,51]. The presented study demonstrated decreased survival of patients aged 70+ years in the combined analyses and in analyses stratified by age and tumor grade, HR status and HER2/neu expression, respectively. Except for the small group of patients with low grade BRC, a major age gradient was seen in all other patient subgroups.

BRC treatment recommendations are essentially based on the prognostic factors examined in this study, i.e. age and stage at diagnosis, tumor grade, HR status and HER2/neu expression[52]. Thus, the data derived from the modelled and stratified analyses may provide clinically relevant information on cancer survival and related excess risks of death for rather distinct but unselected groups of BRC patients with regard to treatment options and prognosis.

The inclusion of patients without local surgery and staging based on clinical examination only aimed at measuring population-based cancer related survival irrespectively of age and stage or for selected subgroups of patients. However, the proportions of these patients were very small for patients with localized or locally or regionally spread BRC and patients below the age of 70 years. For these patients, this study effectively represents survival of patients with definitive surgery.

This study has a number of strengths. The Saarland Cancer Registry provides cancer data with almost complete case ascertainment (regularly estimated above 95%) and follow-up[19,20]. The validity of cancer diagnoses and information on tumor characteristics may be considered as high, as the cancer data were derived from different sources. Detailed clinical information was reasonably complete in 2005-2009, i.e. information on stage, HR status and HER2/neu expression was available in more than 80%, and information on morphology and grade was available for more than 95% of the patients. At first glance, the extent of missing values of the clinical items may appear high. However, in contrast to hospital based registries (covering the patients treated by the respective health care provider instead of a specified source population), population-based registries commonly do not collect clinical items such as stage, HR status, HER2/neu expression at all or do have these data for few patients only.

The use of multiple imputation allowed to overcome the exclusion of substantial numbers of patients with missing values and potential biases resulting from such exclusions in all survival analyses. Some recent studies have shown multiple imputation as a feasible and reasonable method for replacing missing values in datasets with similar levels of missing values[39,53].

It is well known that the completeness of information particularly depends on the duration of the disease and the number of physicians engaged in the treatment of the patients (e.g. invasive examinations to determine the stage of disease may be considered less relevant for patients who will only receive palliative treatment; in this study, the proportions of BRC patients with clinical stage only increased by age and stage accordingly)[54].

Adjusting for different levels of completeness of information across different subgroups of patients with regard to tumor characteristics and duration of follow-up, the chosen multinomial logit model effectively assumed MAR within the different subgroups of patients.

After multiple imputation, the number of patients who contributed survival experience and who could be included into the analyses increased substantially: 7606 patients of the imputed dataset could be included into the “complete” regression model (II) compared to only 4915 patients in the "complete case" scenario. The magnitude and direction of the estimated effects were quite similar if derived from the imputed dataset and the "complete case" analysis, however, the size of the effects generally was somewhat higher in the complete case analysis (data not shown).

The use of period analysis methodology further allowed to derive survival estimates that closely predict survival later observed for the patients diagnosed in the respective calendar period[29,31,32]. This is particularly useful for malignancies with ongoing improvement of cancer survival such as BRC[17].

Although Saarland constitutes only 1.3% of the national population, it is well representative for Germany and its health care system. Age standardized BRC incidence (based on the Europe standard population) in Saarland was 114.6 per 100,000 person years in 2005-2009 [55], and was similar to the incidence in Germany[56]. Age standardized mortality was 28.7 [55] and slightly higher than on the national level[21]. Organized mammography screening started in Saarland at the end of 2006. With ongoing implementation of specialized BRC units [57,58], which started in Saarland in 2004 [59], the database has markedly improved in terms of completeness of information. The proportion of BRC patients with DCO notified tumors was rather small, and the exclusion of these patients from the analyses only had a negligible effect on the derived survival estimates[60].

However, important limitations must be considered as well. The used age ranges divided patients into three broad age categories, which allowed best use of the data in the performed multivariable analyses. Further analyses based on much larger datasets from several German regions would be helpful to evaluate age related cancer mortality more in detail taking into account survival differences that have been observed in the past (e.g. among younger BRC patients aged up to 49 years [61,62]). The use of rather crude stage categories was a trade-off between a comprehensive characterisation of the tumors according to the extent of disease and best possible use of the available data. Furthermore, this study did not include information on administered BRC care, further clinical, socio-demographic or lifestyle factors, which could have helped to better explain to what extent survival differences between patient subgroups (e.g. between patients aged 70 years or older and younger ones) might have been therapy related, or due to other factors, such as co-morbidities, socio-economic status or other determinants.

The underlying population allowed detailed analyses for major tumor characteristics and prognostic factors with sufficient precision. However, some strata (e.g. patients with low grade tumors or metastasized BRC) were small and thus, the derived survival estimates had large standard errors (>5% units). Here, the use of multiple imputation and regression modelling for period analysis provided a valuable tool for evaluating cancer survival. Furthermore, the relative proportions of ductal, lobular and mixed type malignancies required an aggregation of the remaining types, which included both cancers with favourable and very poor prognosis[47].

Despite its limitations, this high resolution study presents survival data of BRC patients from Germany much more in detail than previous studies and therefore extends available age- and stage-specific population-based data on BRC survival. It revealed major effects of tumor grade, HR status and HER2/neu expression on BRC survival on a population level for German patients for the first time. With its high level of detail, this survival study may add clinically relevant information. Based on an unselected population of cancer patients, the study provided important information on cancer survival and cancer related excess mortality for clinically different subgroups of cancer patients, which are not only important for clinicians, but also of major interest for researchers and health care planners. Like previous studies, this study revealed inferior survival of elderly patients aged 70 years or older – and it demonstrated inferior survival consistently across the subgroups of patients with regard to the included clinical factors. Particular efforts should therefore be made to elucidate the reasons for the age gradient in cancer related mortality and to overcome the survival deficits among older patients, who represent one out of three women with a diagnosed BRC. 

## Supporting Information

Appendix S1 Observed (all cause) survival and excess risks of death. (DOC)
