# Supplementary material for: Breast Cancer Survival in Germany: A Population-Based High Resolution Study from Saarland
Source: PLoS One. 2013 Jul 31;8(7):e70680. doi: 10.1371/journal.pone.0070680 (PMC3729832; doi:10.1371/journal.pone.0070680)
Supplement: Appendix S1 — (DOC) [file pone.0070680.s001.doc]

**Appendix S1**

**Observed (all cause) survival and excess risks of death**

Table S1 presents estimates of 5-year observed (all cause) survival and excess risks of death according to age and tumor characteristics. Like for the estimates of relative survival, actuarial methods were used to obtain period estimates of observed survival. [29] The required changes of the used regression models may be found elsewhere. [35]

**Table S1.** Five-year observed survival and excess risk of death of female patients from Saarland with invasive breast cancer (ICD-10 code: C50) estimated for the calendar period 2005-2009 by age and tumor characteristics based on completed datasets

| Characteristic | Category | OS ^a^ | SE | Excess risk ^b^ | 95% CI | p-value | Excess risk ^c, d^ | 95% CI | p-value |
| --- | --- | --- | --- | --- | --- | --- | --- | --- | --- |
|  |  |  |  |  |  |  |  |  |  |
|  |  |  |  |  |  |  |  |  |  |
| Overall |  | 72.7 | 0.7 |  |  |  |  |  |  |
|  |  |  |  |  |  |  |  |  |  |
| Age | 15-49 | 88.2 | 1.2 | 1.00 | REF |  | 1.00 | REF |  |
|  | 50-69 | 84.1 | 0.8 | 1.36 | 1.07-1.73 |  | 1.39 | 1.09-1.78 |  |
|  | 70+ | 56.7 | 1.4 | 3.99 | 3.17-5.01 | <0.001 | 4.31 | 3.42-5.43 | <0.001 |
|  |  |  |  |  |  |  |  |  |  |
| Stage | localized | 84.8 | 1.0 | 1.00 | REF |  | 1.00 | REF |  |
|  | regionally/locally advanced | 69.8 | 1.1 | 2.50 | 2.10-2.97 |  | 2.43 | 2.04-2.89 |  |
|  | distant | 20.0 | 2.3 | 12.79 | 10.52-15.54 | <0.001 | 11.92 | 9.79-14.52 | <0.001 |
|  |  |  |  |  |  |  |  |  |  |
| Morphology ^d^ | invasive ductal | 72.4 | 0.9 | 1.00 | REF |  | 1.00 | REF |  |
|  | invasive lobular | 71.6 | 1.8 | 0.97 | 0.82-1.17 |  | 1.09 | 0.91-1.31 |  |
|  | mixed type | 81.1 | 2.3 | 0.65 | 0.49-0.86 |  | 0.74 | 0.56-0.98 |  |
|  | other | 72.1 | 2.3 | 1.17 | 0.94-1.46 | 0.001 | 1.13 | 0.91-1.40 | 0.055 |
|  |  |  |  |  |  |  |  |  |  |
| Tumor grade ^d^ | low | 86.6 | 2.2 | 1.00 | REF |  | 1.00 | REF |  |
|  | intermediate | 75.0 | 0.8 | 1.66 | 1.12-2.47 |  | 1.60 | 1.07-2.38 |  |
|  | high | 64.5 | 1.4 | 2.54 | 1.70-3.79 | <0.001 | 1.99 | 1.32-3.01 | <0.001 |
|  |  |  |  |  |  |  |  |  |  |
| Hormone receptor status ^d^ | positive (ER+ PgR+) | 76.5 | 0.8 | 1.00 | REF |  | 1.00 | REF |  |
|  | mixed (ER+ or PgR+) | 71.3 | 2.1 | 1.36 | 1.10-1.68 |  | 1.32 | 1.07-1.63 |  |
|  | negative (ER- PgR-) | 58.4 | 2.0 | 2.35 | 2.02-2.74 | <0.001 | 2.09 | 1.77-2.48 | <0.001 |
|  |  |  |  |  |  |  |  |  |  |
| HER2/neu expression ^d^ | negative | 74.2 | 0.8 | 1.00 | REF |  | 1.00 | REF |  |
|  | positive | 69.1 | 1.6 | 1.16 | 0.98-1.37 | 0.079 | 0.97 | 0.82-1.14 | 0.684 |

**NB:** OS: point estimate of 5-year observed survival; SE: standard error of OS; CI: confidence interval; ER: estrogen receptor; PgR: progesterone receptor; REF: reference group; ^a^) except for age group-specific estimates, age standardized estimates were derived using the ICSS weights; ^b^) adjusted for age and stage; ^c^) adjusted for age, stage, morphology, tumor grade, hormone receptor status and HER2/neu expression ("complete" model); ^d^) cases without microscopic verification were excluded; the survival estimates were derived from 10 completed datasets using multiple imputation
